# Specific EEG Changes Associated with Atrophy of Hippocampus in Subjects with Mild Cognitive Impairment and Alzheimer's Disease

**DOI:** 10.1155/2012/253153

**Published:** 2012-02-12

**Authors:** D. V. Moretti, A. Prestia, C. Fracassi, G. Binetti, O. Zanetti, G. B. Frisoni

**Affiliations:** IRCCS Centro S. Giovanni Di Dio Fatebenefratelli, 25125 Brescia, Italy

## Abstract

We evaluated the association between hippocampal atrophy and increase of the EEG markers alpha3/alpha2 relative power ratio in mild cognitive impairment (MCI) and Alzheimer's disease patients. Seventy-nine subjects with MCI and 11 patients with AD underwent EEG recording and MRI scan. The MCI group was subdivided in three subgroups according to growing hippocampal atrophy. The groups were characterized by alpha3/alpha2 relative power ratio. In AD patients group mapped hippocampal regions were computed and related with alpha3/alpha2 power ratio. Results show that the increase of alpha3/alpha2 power ratio is correlated with atrophy of hippocampus both in MCI and in Alzheimer's disease patients. This finding confirms the possible diagnostic role of EEG markers as diagnostic and prognostic factors in patient with prodromal and declared Alzheimer's disease.

## 1. Introduction

Mild cognitive impairment (MCI) refers to the transitional state between the cognitive changes of normal aging and very early dementia [1]. Patients with MCI, who are at high risk of developing Alzheimer disease (AD; [2]), have smaller hippocampal volume than healthy elderly people [3, 4]. Medial temporal lobe (MTL) structures, in particular the hippocampus, show atrophy in the early stages of AD and are potential markers for detecting preclinical AD [5–7]. Moreover, a recent study has demonstrated that atrophy of the hippocampus on MRI in cognitively intact elderly people predicts dementia, in particular of Alzheimer type, during a 6-year followup [8].

Hippocampus is particularly important for memory formation, for attention [9] and for production of EEG rhythmic activity [10, 11]. Lesions of hippocampal synaptic plasticity block the memory-enhancing effects of direct hippocampal stimulation [12, 13]. Further, behavioral stress interferes with synaptic plasticity in the hippocampal formation [14–16]. The associative memories involve the dorsal hippocampus, and a lesion of the area reduces the retrieval of associative tasks [17]. The hippocampal network system seems to be well suited to receive synaptic inputs from both the anterior and posterior thalamic nuclei [18–20], becoming suitable for an association with brain rhythms activity generation.

Recent works showed that in subjects with MCI is present, an increase of high alpha as compared to low alpha band occurs [21, 22]. As a working hypothesis, EEG markers alpha3/alpha2 power ratio could show modifications proportional to the hippocampal atrophy. In the present study the association between hippocampal atrophy and increase of alpha3/alpha2 relative power ratio was investigated in subjects with MCI.

Recent studies have demonstrated that the hippocampus is not a unitary structure from an anatomophysiological point of view [23]. The hippocampus, including strictly speaking subfields CA1–CA4 and the hippocampal formation, including also dentate gyrus, fimbria, subiculum, and parasubiculum, is a highly sophisticated structure. Stimuli coming from the entorhinal cortex are processed by the dentate gyrus, subfields CA4 and CA3, before being projected outside the medial temporal lobe via CA1 or subicular efferent projections. Moreover, in addition to the unsurprising right-left specialization for verbal and visuospatial material [24], some degree of anterior-to-posterior specialization has been shown by fMRI studies [25].

As a consequence, it is conceivable that local structural changes take place in the hippocampus of patients with AD and that different hippocampal subregions are affected in AD Brickman et al. [26], and Shen et al. [27]. Local changes in hippocampal subregions could be detected through a radial atrophy mapping method able to assess group, based on high resolution MRI at 3 Tesla differences [23]. In this study, we tested the hypothesis that the increase of alpha3/alpha2 ratio is related with volumetric differences both in MCI patients and in mapped hippocampal regions in AD patients.

## 2. Materials and Methods

### 2.1. Subjects

For the present study, 79 subjects with MCI and 11 subjects with Alzheimer's disease (AD) were recruited from the memory Clinic of the Scientific Institute for Research and Care (*IRCCS*) of Alzheimer's and psychiatric diseases “Fatebenefratelli” in Brescia, Italy. All experimental protocols had been approved by the local ethics committee. Informed consent was obtained from all participants or their caregivers, according to the Code of Ethics of the World Medical Association (Declaration of Helsinki).

### 2.2. Diagnostic Criteria

#### 2.2.1. MCI Patients

Patients were taken from a prospective project on the natural history of MCI. The project was aimed to study the natural history of nondemented persons with apparently primary cognitive deficits, that is, deficits not due to psychic (anxiety, depression) or physical (hypothyroidism, vit. B12 and folate deficiency, uncontrolled heart disease, and uncontrolled diabetes) conditions. Patients were rated with a series of standardized diagnostic and severity instruments, including the Mini-Mental State Examination (MMSE; [28]), the Clinical Dementia Rating Scale (CDRS; [29]), the Hachinski Ischemic Scale (HIS; [30]), and the Instrumental and Basic Activities of Daily Living (IADL, BADL, [31]). In addition, patients underwent diagnostic neuroimaging procedures (magnetic resonance imaging, MRI) and laboratory testing to rule out other causes of cognitive impairment. These inclusion and exclusion criteria for MCI were based on previous seminal studies [32–38]. Inclusion criteria of the study were all of the following: (i) complaint by the patient, or report by a relative or the general practitioner, of memory or other cognitive disturbances; (ii) Mini-Mental State Examination (MMSE) score of 24 to 27/30, or MMSE of 28 and higher plus low performance (score of 2–6 or higher) on the clock drawing test [39]; (iii) sparing of instrumental and basic activities of daily living or functional impairment steadily due to causes other than cognitive impairment, such as physical impairments, sensory loss, and gait or balance disturbances. Exclusion criteria were any one of the following: (i) patients aged 90 years and older; (ii) history of depression or juvenile-onset psychosis; (iii) history or neurological signs of major stroke; (iv) other psychiatric diseases, epilepsy, drug addiction, and alcohol dependence; (v) use of psychoactive drugs, including acetylcholinesterase inhibitors or other drugs enhancing brain cognitive functions; (vi) current or previous uncontrolled or complicated systemic diseases (including diabetes mellitus) or traumatic brain injuries. All patients underwent (i) semistructured interview with the patient and, whenever possible, with another informant (usually, the patient's spouse or a child of the patient) by a geriatrician or neurologist; (ii) physical and neurological examinations; (iii) performance-based tests of physical function, gait, and balance; (iv) neuropsychological battery assessing verbal and nonverbal memory, attention and executive functions (Trail Making Test B-A; Clock Drawing Test; [40]), abstract thinking (Raven matrices; [41]), frontal functions (Inverted Motor Learning; [42]), language (Phonological and Semantic fluency; Token test; [43]), and apraxia and visuoconstructional abilities (Rey figure copy; [44]); (v) assessment of depressive symptoms by means of the Center for Epidemiologic Studies Depression Scale (CES-D; [45]). Inclusion and exclusion criteria were homogeneous with previous works [46–48]. As the aim of our study was to evaluate the meaning of alpha3/alpha2 power ratio and its associations with structural changes of hippocampus as diagnostic marker of cognitive impairment, we were not interested in this study in the clinical subtype of MCI, that is, amnesic or nonamnesic, single or multiple domains.

#### 2.2.2. AD Patients

The diagnosis of AD was made according to NINCDS-ADRDA criteria [49] and the Diagnostic and Statistical Manual of Mental Disorders IV [50]. Patients were rated with the same series of standardized diagnostic as MCI cohort.

### 2.3. EEG Recordings

All recordings were obtained in the morning with subjects resting comfortably. Vigilance was continuously monitored in order to avoid drowsiness.

The EEG activity was recorded continuously from 19 sites by using electrodes set in an elastic cap (Electro-Cap International, Inc.) and positioned according to the 10–20 International system (Fp1, Fp2, F7, F3, Fz, F4, F8, T3, C3, Cz, C4, T4, T5, P3, Pz, P4, T6, O1, and O2). The ground electrode was placed in front of Fz. The left and right mastoids served as reference for all electrodes. The recordings were used off-line to rereference the scalp recordings to the common average. Data were recorded with a band-pass filter of 0.3–70 Hz and digitized at a sampling rate of 250 Hz (BrainAmp, BrainProducts, Germany). Electrodes-skin impedance was set below 5 kΩ. Horizontal and vertical eye movements were detected by recording the electrooculogram (EOG). EOG activity was recorded with cup electrodes for the control of blinking and eye movements. A cup electrode placed 1 cm above supraorbital ridge registered the vertical EOG. It was referred to another electrode placed 2 cm below suborbital ridge of the right eye. The left and right horizontal EOG channels were collected from two electrodes at the left and the right lateral canthus. These electrodes were referred to an electrode placed at the glabella.

The recording lasted 5 minutes, with subjects with closed eyes. Longer recordings would have reduced the variability of the data, but they would also have increased the possibility of slowing of EEG oscillations due to reduced vigilance and arousal. EEG data were then analyzed and fragmented off-line in consecutive epochs of 2 seconds, with a frequency resolution of 0.5 Hz. The average number of epochs analyzed was 140 ranging from 130 to 150. The EEG epochs with ocular, muscular, and other types of artifacts were discarded. Signals higher than 100 uV were discarded as artifact.

### 2.4. Analysis of Individual Frequency Bands

Frequency bands were determiner on an individuale basis, because of the variability of the brain rhythms with age and diseases. A digital FFT-based power spectrum analysis (Welch technique, Hanning windowing function, and no phase shift) computed the power density of EEG rhythms with a 0.5 Hz frequency resolution, ranging from 2 to 40 Hz. Two anchor frequencies were selected according to literature guidelines (47), which are the transition theta/alpha frequency (TF) and the individual alpha frequency (IAF) peak. As previously mentioned, the TF marks the transition frequency between theta and alpha bands, and it represents an estimate of the frequency at which theta and alpha spectra intersect. We computed the TF as the minimum power in the alpha frequency range since our EEG recordings were performed at rest. The IAF represents instead the frequency with the maximum power peak within the extended alpha range (5–14 Hz). The TF and IAF could be clearly identified in 99 MCI subjects whose EEG data were then statistically analyzed. Based on the TF and IAF, we estimated for each subject the frequency band range as follows: delta from TF-4 to TF-2, theta from TF-2 to TF, low alpha (alpha1 and alpha2) from TF to IAF, and high alpha band (or alpha3) from IAF to IAF + 2. Alpha1 and alpha2 band were computed for each subject as follows: alpha1 from TF to the middle point of the TF-IAF range and alpha2 from this middle point to IAF peak [46]. We found that the bandwidth in alpha1 and alpha2 bands was different among the groups. In no vascular damage and severe vascular damage groups, it was slightly narrower (1.48 and 1.53 Hz, resp.) than in mild and moderate vascular damage groups (1.7 and 1.87 Hz, resp.). We performed a statistical analysis to test if this difference was significant among groups in these frequency bands. It was not the case, since the analysis did not show a main group significant effect (*P* = 0.06). Finally, in the frequency bands so determined, we computed the relative power spectra for each subject. Relative power density for each frequency band was computed as the ratio between the absolute power and the mean power spectra from 2 to 40 Hz. The relative band power at each band was defined as the mean of the relative band power for each frequency bin within that band.

### 2.5. MRI Scans

MRI scans were acquired with a 1.0 Tesla Philips Gyroscan at the Neuroradiology Unit of the Città di Brescia hospital, Brescia. The following sequences were used to measure hippocampal volumes: a high-resolution gradient echo T1-weighted sagittal 3D sequence (TR = 20 ms, TE = 5 ms, flip angle = 30°, field of view = 220 mm, acquisition matrix = 256 × 256, and slice thickness = 1.3 mm) and a fluid-attenuated inversion recovery (FLAIR) sequence (TR = 5000 ms, TE = 100 ms, flip angle = 90°, field of view = 230 mm, acquisition matrix = 256 × 256, and slice thickness = 5 mm).

#### 2.5.1. MCI Patients

Hippocampal and white matter hyperintensities (WMHs) volumes were obtained for each subject. The hippocampal boundaries were manually traced on each hemisphere by a single tracer with the software program DISPLAY (McGill University, Montreal, Canada) on contiguous 1.5 mm slices in the coronal plane. The starting point for hippocampus tracing was defined as the hippocampal head when it first appears below the amygdala, the alveus defining the superior and anterior border of the hippocampus. The fimbria was included in the hippocampal body, while the grey matter rostral to the fimbria was excluded. The hippocampal tail was traced until it was visible as an oval shape located caudally and medially to the trigone of the lateral ventricles [51, 52]. The intraclass correlation coefficients were 0.95. White matter hyperintensities (WMHs) were automatically segmented on the FLAIR sequences by using previously described algorithms [51, 52]. Briefly, the procedure includes (i) filtering of FLAIR images to exclude radiofrequency inhomogeneities, (ii) segmentation of brain tissue from cerebrospinal fluid, (iii) modelling of brain intensity histogram as a gaussian distribution, and (iv) classification of the voxels whose intensities were ≥3.5 SDs above the mean as WMHs [51, 52]. Total WMHs volume was computed by counting the number of voxels segmented as WMHs and multiplying by the voxel size (5 mm^3^). To correct for individual differences in head size, hippocampal and WMHs volumes were normalized to the total intracranial volume (TIV), obtained by manually tracing with DISPLAY the entire intracranial cavity on 7 mm thick coronal slices of the T1-weighted images. Both manual and automated methods user here have advantages and disadvantages. Manual segmentation of the hippocampus is currently considered the gold standard technique for the measurement of such complex structures. The main disadvantages of manual tracing are that it is operator dependent and time consuming. Conversely, automated techniques are more reliable and less time consuming, but may be less accurate when dealing with structures without clearly identifiable borders. This, however, is not the case for WMHs which appear as hyperintense on FLAIR sequences.

Left and right hippocampal volumes were estimated and summed to obtain a total volume (individual) of both anatomical structures. Hippocampal total volume has been divided in tertiles obtaining three groups. In each group, hippocampal volume has been computed.

#### 2.5.2. AD Patients: Radial Atrophy Mapping

In AD patients, the 3D parametric surface mesh models were created from the manual tracings of hippocampal boundaries [53, 54]. This procedure allows measurements to be made at corresponding surface locations in each subject, which are then compared statistically in 3D [53, 54]. To assess hippocampal morphology, a medial curve was automatically defined as the 3D curve traced out by the centroid of the hippocampal boundary in each image slice. The radial size of each hippocampus at each boundary point was assessed by automatically measuring the radial 3D distance from the surface points to the medial curve defined for individual's hippocampal surface model.

### 2.6. Statistical Analysis and Data Management

#### 2.6.1. MCI Patients

The analysis of variance (ANOVA) has been applied as statistical tool. Greenhouse-Geisser correction and Mauchly's sphericity test were applied to all ANOVAs. Preliminarily, the significant differences among groups in demographic variables, (age, education, and MMSE score) and morphostructural characteristics, (hippocampal and white matter hyperintensities, WMHs, and volume), were evaluated (Table 1). In order to avoid a confounding effect, subsequent ANOVAs were carried out using age, education, MMSE score, and WMHs as covariates. Duncan's test was used for post-hoc comparisons. For all statistical tests, the significance level was set at *P* < 0.05.

Two separate ANOVAs were performed. The first analysis was performed in order to verify the difference of hippocampal volume among groups. The second ANOVA was performed in order to check differences in alpha3/alpha2 relative power ratio in the three MCI subgroups ordered by decreasing tertile values of the hippocampal volume. In each ANOVA, group was the independent variable, the frequency ratios were the dependent variable.

#### 2.6.2. AD Patients

In AD patients, the radial atrophy mapping was chosen because it is more suitable to study subregional volume of the hippocampus. Correlation maps between EEG rhythms and hippocampal surface were computed. The correlation analysis between EEG rhythms and hippocampal volume was performed only in 11 AD subjects; we otherwise compared hippocampal gray matter distribution maps between normal controls and AD patients in order to verify that the AD found correlations between EEG rhythms and hippocampal regions were present in areas where AD is more atrophic than normal subjects.

**Figure 1:**
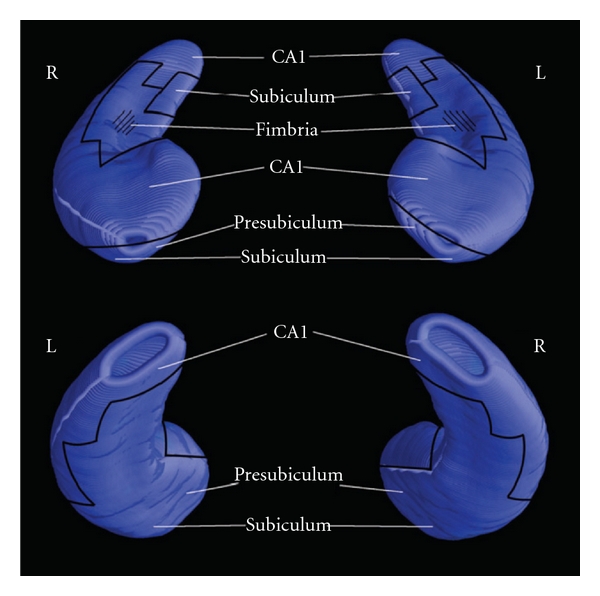
Cytoarchitectonic subregions mapped on blank MR-based models of the hippocampal formation of a healthy subject.

The correlation maps were generated on 3D models of the hippocampal formation where the dorsal and ventral surfaces can be appreciated. Zones with significant correlations were mapped onto the models based on an atlas where these are shown together with the corresponding MR sections ([55, 56]; Figure 1). The correlations and the associated *P* value maps were plotted onto a colour-coded model of the hippocampal surface. The statistical test for the correlations was computed using linear regression at each surface vertex on the hippocampus [54]. A surface point significance threshold of *P* < 0.05 was used to visualize the regional specificity of gray matter changes in the cortex. Set level correction for multiple comparisons was carried out by permutation testing at threshold of *P* = 0.05. Permutation tests are based on measuring the total area of the hippocampus with suprathreshold statistics, after setting the threshold at *P* < 0.05. To correct for multiple comparisons and assign an overall *P* value to each p map permutation, tests were used to determine how likely the observed level of significant atrophy (proportion of suprathreshold statistics, with the threshold set at *P* < 0.05) within each p map would occur by chance. The number of permutations N was chosen to be 100,000, to control the standard error SEp of omnibus probability *P*, which follows a binomial distribution B(*N*, p) with known standard error. When *N* = 8, 000, the approximate margin of error (95% confidence interval) for p is around 5% of p. Both left and right hippocampal volumes were investigated in AD patients given the superior well-known left hemispheric involvement in declared dementia. The analysis on the AD subjects was conducted to verify the reliability of the alpha3/alpha2 ratio as factor associated with conversion of a subpopulation of MCI subjects in Alzheimer's disease. This results need further confirmation in a larger size population to strength the statistical power of the analysis. Of note, a larger population could be permitted to detect more precisely the volume ranges within the hippocampal subregions and their correlation with the EEG marker, ruling out other possible associations.

In the two groups of patients, MCI and AD patients, the MRI analysis method performed was different: volumetric analysis for MCI and radial atrophy mapping for AD patients. These different methods need different statistical approach in order to obtain more reliable results.

## 3. Results

### 3.1. MCI Patients

In this study, we were interested in morphofunctional (MRI-EEG) association and not in neuropsychological issues. MMSE values were provided as a general marker of the entity of the cognitive decline of patients.

Table 1 summarizes the ANOVA results of demographic variables, that is, age, education, MMSE score, and morphostructural characteristics, that is, hippocampal, and white matter hyperintensities volume in the whole MCI cohort as well as in the three subgroups in study. Significant statistical results were found in hippocampal volume (respectively, *F*_2,76_ = 157.27; *P* < 0.00001 and *F*_2,76_ = 132.5; *P* < 0.00001). Duncan's post-hoc test showed a significant increase (*P* < 0.01) in all comparisons.  Table 2 shows the results of alpha3/alpha2 ratio in the groups based on the decrease of hippocampal volumes. ANOVA results revealed significant main effect group in alpha3/alpha2 ratio for hippocampal (*F*_2,76_ = 3.38; *P* < 0.03) decreasing volume.

### 3.2. AD Patients

**Figure 2:**
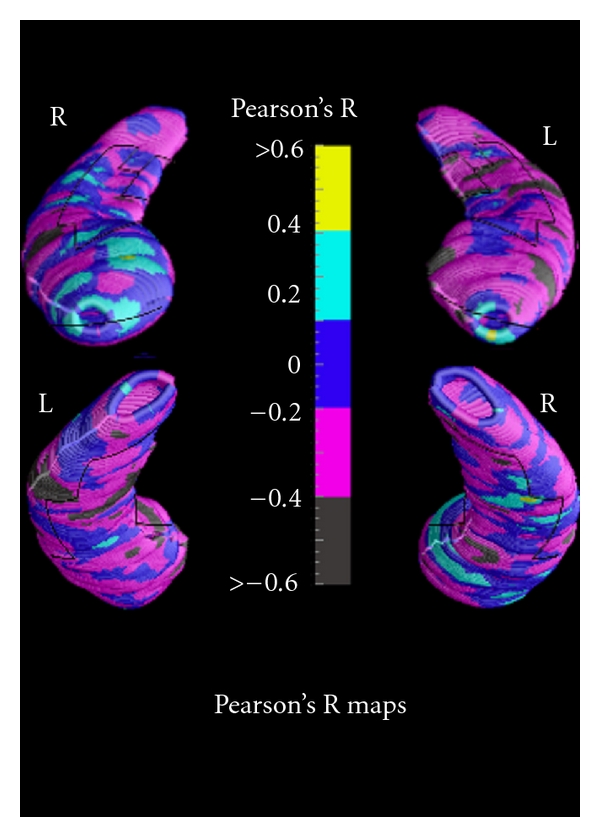
Correlation between alpha3/alpha2 power rhythm ratio and volumes of hippocampal subregions in AD patients.

Table 3 summarizes sociodemographic characteristics, MMSE scores, and alpha3/alpha2 power ratio in AD cohort.  Figure 2 shows correlations between alpha3/alpha2 rhythms ratio and hippocampal volumes in AD patients. Negative significant associations are found between same areas of AD left hippocampus and alpha3/2 EEG rhythms. Correlations in right hippocampus resulted in being not significant at permutation testing (*P* > 0.75  *in* *both* *cases). There was no statistical difference considering the alpha2 or alpha3 spectral power alone.*

## 4. Discussion

### 4.1. Preliminary Considerations

The findings of the present study permit to identify a reliable association between an EEG index (alpha3/alpha2 power ratio) and hippocampal atrophy. This EEG marker shows his reliability both in MCI and AD subjects, suggesting that it could identify some MCI subjects prone to conversion in AD. The principal limitations of the study are (1) the small size of the AD group; (2) the lack of a normal control group. These caveats need to be addressed by future studies performing a correlation analysis approach.

### 4.2. Alpha3/Alpha2 Ratio: Possible Relationship with Hippocampal Volume and Physiological Meaning in MCI and AD Patients

The increase of alpha3/alpha2 ratio is associated with the decrease of hippocampal volume, confirming previous results of our group showing that the increase of high alpha is related to hippocampal atrophy in MCI patients [21, 22]. The results show that in AD patients, increase of alpha3/alpha2 power ratio is correlated with the decrease of left hippocampal gray matter volumes. In particular, hippocampal areas involved in correlation are presubiculum, dorsal and ventral subiculum, CA2-3 sectors of the body, CA1 mesial, and lateral portion of the head. The prevalence of the modification of EEG rhythms in the left hemisphere in patients with AD was found also in a recent EEG coherence study [57]. Indeed, in this study, pathologic changes of connectivity are significant on the frontotemporal region of the left hemisphere, but not on the right. Our findings confirm the asymmetry between left and right planum temporale previously shown in patients with AD [58]. A large body of literature has demonstrated the crucial role of the left hemisphere in semantic associative encoding [59] and of the left hippocampal-medial prefrontal pathways [60, 61].

The increase of high alpha synchronization has been found in internally-cued mechanisms of attention, associated with inhibitory top-down processes [62], acting as filter to irrelevant information. This filter activity could be carried out by hippocampus. Indeed, a recent work has demonstrated that the mossy fiber (MF) pathway of the hippocampus, connecting the dentate gyrus to the autoassociative CA3 network, is controlled by a feedforward circuit combining disynaptic inhibition with monosynaptic excitation. Analysis of the MF-associated circuit revealed that it could act as a highpass filter [63, 64].

The loss of inhibitory mechanism at hippocampal level impairs the filter function of hippocampus. The increase in cortico-subcortical inputs to hippocampal formation determines an increase of memory retrieval effort in long-term memory system and dysregulation of divided attention, in particular when multiple stimuli have to be processed [65–67] inducing behavioural dysfunction as well as subsequent memory deficits. The exchange of information between memory and attentive systems has been associated upper alpha band desynchronization [68, 69]. The synchronization of high alpha power has been demonstrated to be involved in top-down cognitive processes. This finding could suggest that there is an the attempt to focus attention on highly selective aspect to prevent interference of irrelevant stimuli (top-down process) in order to maintain a good memory performance [62]. Recently, we suggest that MCI subjects could fall in a “hyperattentive state” during the course of disease. Our results confirm this hypothesis, extending those findings to AD patients [70].

### 4.3. Hippocampal Formation and Alpha3/Alpha2 Ratio: Possible Network Interactions

A possible explanation should strongly consider that our results are obtained in an idling state. So, the discussion of the results has to address the default state of the brain. In this point of view, in a normal default state, large cell assemblies cooperate to keep an extensive network. This state is represented by the low alpha rhythm, typical of the EEG idling state. The increase of the alphae3/alpha2 power ration could suggest in MCI and AD patients the prevalence of smaller cell assemblies in the default state, due to synaptic disfunction or brain atrophy. Of note, seminal studies have demonstrated that large cell assemblies oscillates in low frequencies, whereas smaller cell assemblies develop higher frequencies [71].

The specific involvement of alpha rhythm could suggest that the hippocampal atrophy in AD is linked to functional changes in a broader network. Of note, the hypometabolism and atrophy of posterior cingulate/retrosplenial and medial temporal cortex pathway, strictly connected with both hippocampus and visual cortex, as well as with low alpha rhythm generation, are well demonstrated in AD [72]. So, the prevalence of high alpha could underlie the disruption of extensive synaptic connection deriving in the formation of smaller cell assemblies. The impairment of the network could explain memory and cognitive symptoms of AD beyond the hippocampal atrophy itself [70].

## 5. Conclusion

Our findings confirm the possible diagnostic role of EEG activity when integrated with morphostructural measures in patients with AD.

## Figures and Tables

**Table 1:** Mean values ± standard deviation of sociodemographic characteristics, MMSE scores, white matter hyperintensities, and hippocampal volume measurements in MCI cohort.

	MCI cohort	Group 1	Group 2	Group	*P* value (ANOVA)
Number of subjects (f/m)	79 (42/37)	27 (14/13)	27 (15/12)	25 (13/12)	
Age (years)	69.2 ± 2.3	66.8 ± 6.8	69.4 ± 8.7	71.5 ± 6.9	0.1
Education (years)	7.7 ± 0.8	8.3 ± 4.5	6.7 ± 3.1	8.2 ± 4.6	0.2
MMSE	27.1 ± 0.4	27.5 ± 1.5	27.4 ± 1.5	26.6 ± 1.8	0.1
Individual hippocampal volume (mm^3^)	4889.8 ± 962.4	5809.6 ± 314.2	4969.4 ± 257.6	3890.1 ± 551.4	0.00001
White matter hyperintensities (mm^3^)	3.8 ± 0.5	3.2 ± 2.8	4.2 ± 3.8	4.1 ± 3.6	0.7

**Table 2:** Relative alpha3/alpha2 relative power band ratios according to hippocampal volumes.

Hippocampal volume	Alpha3/alpha2 ratio (*μ*v^2^)	*P* value
Group1	1.04 ± 0.11	0.03
Group2	1.11 ± 0.15	
Group3	1.12 ± 0.14	

**Table 3:** Mean values ± standard deviation of sociodemographic characteristics, MMSE scores, and alpha3/alpha2 power ratio in AD cohort.

	AD
Number of subjects (f/m)	11 (6/5)
Age (years)	76.2 ± 2.3
Education (years)	4.6 ± 0.9
MMSE	21.3 ± 2.5
Alpha3/alpha2 ratio	1.5
